# Late initiation of antenatal care and associated factors among pregnant women in Jimma Zone Public Hospitals, Southwest Ethiopia, 2020

**DOI:** 10.1186/s12913-022-08055-6

**Published:** 2022-05-12

**Authors:** Fetlework Tadele, Nigusu Getachew, Kelemu Fentie, Demuma Amdisa

**Affiliations:** 1 Jimma University Medical Center, Jimma, Ethiopia; 2 Department of Health Policy and Management, Faculty of Public Health, Institute of Health, Jimma University, Jimma, Ethiopia; 3 Department of Health Promotion and Behavior, Faculty of Public Health, Institute of Health, Jimma University, Jimma, Ethiopia

**Keywords:** Antenatal care, Late initiation, Pregnant women, Jimma zone public hospitals

## Abstract

**Background:**

Late antenatal care initiation is linked to a higher risk of maternal death. Women who do not start ANC at an early stage may experience the effects of pregnancy-related health difficulties, as well as long-term health issues and pregnancy complications. Therefore, our study aimed to determine the prevalence of late initiation of antenatal care and associated factors among pregnant women in Jimma Zone public Hospitals.

**Methods:**

A facility-based cross-sectional study design was employed in Jimma zone public hospitals from February 1 up to 30 March 2020 and 409 pregnant women were participated in the study by using a systematic random sampling method. Structured questionnaire was used to collect data that contain socio demographic variables, socio cultural variables, pregnancy related factors and predisposing factor related variables. The data was entered into EPI data version 3.1 and exported to SPSS version 20 for statistical analysis. Binary and multivariable logistic regression analysis were performed by using 95%CI and significance was declared at *P* < 0.05.

**Result:**

Forty-eight percent of pregnant women were initiated their first ANC late. Primary education (AOR = 0.242; 95% CI, 0.071–0.828) and college diploma and above was (AOR = 0.142; 95% CI, 0.040- 0.511), mothers with an unplanned pregnancy (AOR = 11.290; 95%CI, 4.109–31.023), time taken to arrive the health facility greater than sixty (60) minutes (AOR = 8.285; 95% CI, 2.794–24.564) and inadequate knowledge about ANC service (AOR = 4.181; 95%CI, 1.693–10.348) were associated with late first Antenatal care initiating.

**Conclusion:**

The prevalence of late initiation of ANC still remains a major public health concern in the study area. Level of education, unplanned pregnancy, distance from house to health facility, and lack of understanding about ANC services were all found to be significant variables in late ANC starting. As a result, healthcare workers can provide ongoing health education on the need of starting antenatal care visits early to avoid unfavorable pregnancy outcomes by considering all identified factors.

## Introduction

Antenatal care (ANC) is a type of care given to pregnant women and is considered a key maternal service in improving a wide range of health outcomes for women and newborns. The Antenatal Care (ANC) model recommended by the World Health Organization (WHO) calls for a minimum of eight ANC visits, with the first one occurring during the first trimester [1]. ANC initiation period is defined differently in different countries; European countries consider it a late ANC initiation if it occurs after the first trimester, while Pakistan considers it a late ANC initiation if it occurs after 24 weeks of gestation [2, 3]. Late ANC initiation, on the other hand, is defined in Ethiopia as a pregnant woman visiting for the first time after 16 weeks of pregnancy [4].

Implementing appropriate ANC services and evidence-based techniques during ANC at the suggested period can benefit both the mother and the fetus. They also promote maternal nutrition, give health education, and help to avert obstetric emergencies and other life-threatening situations. This is also an appropriate time for women to begin planning for birth and newborn care [5–8].

In the year 2001, the WHO issued guidance on a new model of ANC called goal-oriented or focused ANC, for implementation in developing countries [9]. In low-risk pregnancies, WHO recommends four antenatal care visits with evidence-based content prescribed for each: the first during the first trimester; the second around week 26; the third around week 32; and the fourth and final visit between weeks 36 and 38 [9, 10].

The late ANC initiation magnitude is different for countries. The lowest among European countries was 2% for Poland, and the highest was 33% for Malta [11]. In Pakistan, 71% of women received late antenatal care [12]. According to the Ethiopian Demographic Health Survey (EDHS, 2016), 80% of women had their first ANC visit after 16 weeks of gestational age [5]. Late initiation of ANC is a significant risk factor for maternal mortality. Women who do not begin ANC at an early stage may experience the effects of pregnancy-related health conditions, as well as long-term health problems and complications related to pregnancy [7, 13].

Every day, over 800 women die from complications connected to pregnancy and childbirth around the world, with Sub-Saharan Africa, which includes Ethiopia, accounting for over 66% of those deaths. According to the World Health Organization (WHO), 11,000 maternal deaths occurred in Ethiopia in 2015 during pregnancy, childbirth, and the postpartum period [14]. Postpartum hemorrhage, sepsis, pre-eclampsia, eclampsia, and delivery complications are the leading causes of maternal mortality in Ethiopia [2]. Despite the fact that the majority of maternal deaths are preventable, many women lack access to evidence-based interventions including antenatal care (ANC) during pregnancy and related services during labor and the postpartum period due to poverty, a lack of awareness, and cultural aspects [11].

One of the strategies used to reduce maternal mortality was timely and high-quality antenatal care [8]. The predictors associated with late initiation of antenatal care varied across studies, including residence, education of the pregnant woman, husband's occupation, parity and being planned, women who had no previous obstetrical complications, and those who lived far away from health care facilities, among others [8, 11, 12]. In order to decrease child and maternal mortality, it is critical to know the time of the first ANC visit for pregnant women and the factors affecting it. Therefore, this study aimed to measure the prevalence and factors associated with late initiation of ANC at Jimma Zone Public Hospitals.

## Method and materials

### Study setting and design

A facility-based cross-sectional study design was carried out from February 1 to 30, 2020 in Jimma zone public hospitals. The zone has eight public hospitals, two private hospitals, and 120 health centers. Of the eight governmental hospitals, one is a referral hospital, three are general hospitals, and four are primary hospitals. The source and study population were all pregnant women who attended selected public hospitals of the ANC clinic and participant who were selected for the study during the data collection period from the sampled hospitals respectively. All pregnant women who were initiated into ANC were included, and who were severely ill and who attended their first ANC visits in other health facilities were excluded.

### Sample size determination and sampling technique

**Fig. 1:**
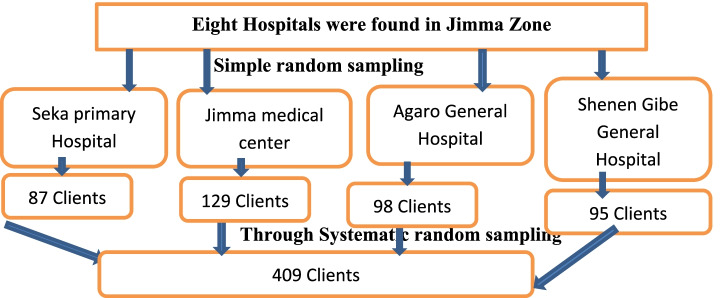
Schematic presentation of sampling procedure

A single population proportion formula was used assuming a 95% confidence interval and a 59% prevalence (P) of receiving ANC [15] and a precision of 5% between the sample and the 10% non- response rate, thus a total of 409 pregnant women were required for the study. Simple random sampling techniques were used to select the hospitals. Of the total of eight hospitals found in the Jimma zone, four (Jimma University medical center, Agaro general hospital, Seka primary hospital, and Shenen Gibe general hospital) were selected for the study. Finally, proportional allocation to sample size of each hospital was done, all eligible study participants were selected from each hospital till the allocated sample size was reached by systematic random sampling with kth interval values. (Fig. 1).

### Data collection tools and personnel

An interviewer-administered structured questionnaire was adapted through reviewing existing literature which encompasses information related to socio-demographic characteristics, pregnancy-related variables, socio-cultural variables, and predisposing factors [16, 17]. Data were collected by trained data collectors using pretested and structured questioners. The questionnaires were prepared in English, then translated into the languages of both Afaan Oromo (the language spoken by the local residents) and Amharic versions (the official language of Ethiopians), and retranslated back to English by experts to ensure consistency. Face-to-face interviews were used to collect data; gestational age was estimated by asking about the last normal menstrual period, and if respondents were unable to recall their gestation age, further informed consent was obtained to access medical records. Two days of training were given by the principal investigator, which focused on the objective of the study to create a common understanding of the questionnaires. A pretest was conducted among 20 pregnant women in the Limu Genet Primary hospital. After the pretest, the necessary correction was made.

### Study variables

Dependent Variable was late initiation of ANC and the independent variables were Socio-demographic variables such as (age, educational status, marital status, and occupation of respondents’, house Income and residence), Socio-cultural variables (religion, family support, advice from significant others, Decision-making and Husbands Educational status), Pregnancy related variables (number of delivery, gravidity, abortion history), Types of pregnancy and means of recognizing pregnancy and Predisposing factors (Knowledge of pregnant women related to ANC visits, and pregnancy related health problem). 

### Operational definitions

Antenatal care:-is the care given to pregnant women so that they have safe pregnancy and healthy baby.

#### Late initiation of ANC

Pregnant women were considered late ANC initiated at first visit, when they came to health facility after 16 weeks of gestational age.

#### Inadequate knowledge

Respondents were categorized as inadequate knowledge about ANC service, if they scored below to the mean knowledge score questions of about ANC service (34), otherwise adequate knowledge.

#### Planned pregnancy

A pregnancy which is consciously desired and planned by a couple

### Data processing and analysis

The completed questionnaire was coded and entered into a data entry template in EPI-DATA version 3.1, then exported to SPSS version 20 for analysis. Descriptive statistics like frequencies, cross-tabulation, graphs, and percentages were employed. The goodness of fit was checked with the Hosmer–Lemeshow test (*p* = 0.35). Multicollinearity was checked by examining the variance inflation factor. In the bi-variable logistic regression analysis, *p*-values of less than 0.25 were used to select the candidate variables for multivariable logistic regression analysis. An adjusted odds ratio (AOR) with a 95% CI was used to determine the predictor of the outcome variable independently and to show the strength of an association. A *p*-value of less than 0.5 were considered as statistically significant**.**

## Result

### Socio-demographic characteristics of the study participants

A total of 409(100%) clients were included in the interview, yielding 100% response rate. The mean age was 27 ± 4.22 years. Among the study participant 84.6% of them were married, 10.8% were single, and more than one-third of the respondents, 36.4%, were housewives. The majority of the respondents, 68.45% were urban residents and 70.9% had 1500 ETB (44.12 US dollars) (Table 1).

**Table 1:** Socio-demographic characteristics of the pregnant women to assess late initiation of ANC among pregnant women, Jimma Zone Public Hospitals, Ethiopia, 2020

Variables(*n* = 409)	Frequency(n)	Percentage (%)
**Age group**		
15–19	3	0.7
20–24	90	22.0
25–29	214	52.3
30–34	78	19.1
35 and above	24	5.9
**Religion**		
Muslim	189	46.2
Orthodox	134	32.8
Protestant	68	16.6
Catholic	18	4.4
**Marital status**		
Married	346	84.6
Single	44	10.8
Widowed	11	2.7
Divorced	8	1.9
**Occupational**		
House wife	173	42.3
Government.employee	91	22.2
Merchant	85	20.8
Farmer	25	6.1
Student	35	8.6
**Educational status**		
Only read and write	90	22.0
Can read and write	53	13.0
primary school (1–8)	82	20.0
secondary school (9–12)	88	21.5
college diploma & above	96	23.5
**Educational status of husband**		
Only read and write	48	11.7
can read and write	58	14.2
primary school(1–8)	77	18.8
secondary school(9–12)	87	21.3
college diploma and above	139	34
**Residence**		
Urban	280	68.5
Rural	129	31.5
**HH income**		
≤ 1000 ETB(≤ 29.41US$)	67	16.4
1000-1500ETB(29.41–44.11US$)	52	12.7
≥ 1500 ETB(≥ 44.11US$)	290	70.9
**Husband has another wife**		
Yes	101	24.7
No	308	75.3
**Time taken to health facility**		
< 60 min	292	71.4
≥ 60 min	117	28.6
**Occupational status of Husband**		
farmer	70	17.1
government employee	125	30.6
private employee	121	29.6
merchant	79	19.3
student	11	2.7
others	3	0.7

### Timing of first ANC booking of the study participants

**Fig. 2:**
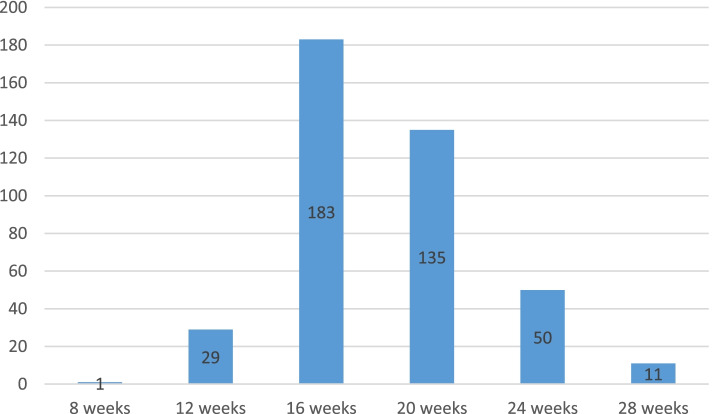
First ANC distribution of pregnant women by month attending ANC in. Jimma Zone Public Hospitals, Ethiopia, 2020

The prevalence of pregnant women who has late initiated their first ANC (after 16 week) age was 48%. Among the pregnant women, 21.5% reported the reason for the specific time for the first ANC visit. They did not know the right time and its purpose. Among the participants 25.6% of the respondents who had been to the late initiation of ANC were between the age groups of 25 and 29 years old. Pregnant women attend their first ANC was 44.7% at the recommended time (16 weeks) (Fig. 2).

### Knowledge of study participant on ANC Service

Out of 409 respondents, 57% and 39% were to prevent potential health problems and regular medical and nursing care during ANC contact respectively. Pregnant women have adequate knowledge, 139(34%), because they obtain the above mean score: 45.83%. Of the total participants, 270 (66%) respondents have inadequate knowledge because they obtained below the mean score of ANC and the mean score of < 45.83% (Table 2).

**Table 2:** Knowledge of pregnant women on ANC, Jimma Zone Public Hospitals, Ethiopia, 2020

Variables		Category	Number	Percent
**ANC definition**	Regular medical and nursing care recommended for woman during pregnancy	Yes	161	39.4%
		No	248	60.6%
	Treat and prevent potential health problems throughout the course of the pregnancy	Yes	233	57.0%
		No	176	43.0%
	Helps in promoting healthy lifestyles that benefits both mother and child	Yes	133	32.5%
		No	276	67.5%
Is antenatal care necessary to pregnant women		Yes	401	98.0%
		No	8	2.0%
**Importance of ANC**	To know the condition of baby	Yes	143	35.0%
		No	266	65.0%
	To know the health of mother	Yes	131	32.0%
		No	278	68.0%
	To avoid complication	Yes	118	28.9%
		No	291	71.2%
	For safe delivery	Yes	336	82.2%
		No	73	17.9%
	It reduces maternal and infants mortality and morbidity	Yes	29	7.1%
		No	380	92.9%
First antenatal check-up done during the first 4 months		Yes	236	57.7%
		No	173	42.3%
Pregnant women need at least five ANC visit?		Yes	239	58.4%
		No	170	41.6

### Predisposing and socio-cultural factors among pregnant women regarding their health care seeking to attend ANC

One-third of the pregnant women (31.3%) responded that they had ANC follow-up during the preceding pregnancy and about (34.2%) participants were responded as experienced a health problem during the preceding pregnancy. Among the participants 64.8% of them were participated in decision-making process about their health care were decided jointly with husband followed by women alone was 28.1%. (Table 3).

**Table 3:** Predisposing factors and socio cultural factors among pregnant women regarding their health care seeking attending ANC in Jimma Zone Public Hospitals, Ethiopia, 2020

Variables	Variables	Frequency	Percent
Decision maker regarding women health care	Women alone	115	28.1
	Husband	27	6.6
	Jointly	267	65.3
Decision maker about current ANC using	Women alone	81	19.8
	Husband	28	6.8
	Jointly	292	71.4
	Others	2	0.5
Have ANC follow up during preceding pregnancy	Yes	128	31.3
	No	281	68.7
Experienced health problem in preceding pregnancy	Yes	140	34.2
	No	269	65.8

### Factors significantly associated with late initiation of antenatal care

Women who reported arriving at the health facility in more than sixty (60) minutes were more than eight times more likely to start ANC recently than those who reported arriving in less than sixty minutes (AOR = 8.26455; 95%CI, 2.929–25.514). Pregnant women who perceived that the current pregnancy was not wanted or unplanned (AOR = 11.290; 95%; CI, 4.109–31.023) were 11 times more likely to start their ANC late compared to those women who wanted the pregnancy. Pregnant women who have inadequate knowledge about ANC service have been (AOR = 4.186; 95%CI, 1.693–10.348) 4 times more likely to start their ANC lately than those who have adequate knowledge. Pregnant mothers who had two children (AOR = 5.964; 95% CI, 1.733–20.526) were six times more likely to begin ANC late than those who had three or more children (Table 4).

**Table 4:** Multivariate analysis for magnitude and factors affecting late initiation of ANC service utilization among antenatal care attendants in Jimma Zone Public Hospitals, Ethiopia, 2020

*Variables*	*Outcome variable*		*COR(95% CI)*	*AOR(95% CI)*
	Late attendants	Early attendants		
*Educational status*				
* Cannot read and write*	69(76.7%)	21(23.3%)	1	1
* Primary*	31(37.8%)	51(62.2%)	0.185(0.095,0.359)	0.242(0.071,0.828)*
* College diploma and above*	29(30.2%)	67(69.8)	0.132(0.68,0.253)	0.142(0.040,0.511)*
*Planned pregnancy*				
* Yes*	82(31.5%)	180(68.7%)	1	1
* No*	114(77.6)	33(22.4)	7.583(4.753,12.099)	11.290(4.109,31.023)**
*Time taken to health facility*				
< *60 min*	100(34.2%)	192(65.8%)	1	1
≥ *60 min*	96(82.1%)	21(17.6%)	8.777(5.164,14.918)	8.26455(2.929,25.514)**
* Knowledge on ANC Adequate knowledge*	96(35.5%)	174(64.4)%	1	1
* Inadequate knowledge*	100(71.9%)	39(28.1%)	7.583(4.753,12.99)	4.186(1.693,10.348)*
*Number of parity*				
* Twice*	29(69.0%)	13(31.0%)	2.839(1.201,6.710)	5.964(1.733,20.526)*
* Three and above*	22(44.0%)	28(56.0%)	1	1

Key; **statically significant, (*p* < 0.001), *statically significant, (*p* < 0.05), COR, crude odds ratio, *AOR* adjusted odds ratio, 1 reference category, Max, VIF = 1.06(no multicollinearity: at VIF < 5)

## Discussion

Our study pointed out that 48% respondents initiated first ANC contact beyond the recommended time. That is almost consistent with the study done in Adigrat Town and Tigray, Ethiopia, (51.8%) [17], and Dilla Town governmental health institutions (50.3%) [18].

The prevalence of pregnant women who initiated late ANC in Jimma Zone (48%) was higher than in Debre Markos town, North West Ethiopia (33.4%) [11].This might be due to socio-cultural differences and difference in awareness creation regarding ANC initiation and difference in accessibility of health facilities. It is also higher than the report of the study in Cameroon (44.0%) [19]. This might be due to the difference in socio-economic level attention and educational background among the study populations in the case of Cameroon.

In the current study the prevalence of late ANC in pregnant women was lower than in Arbaminch town (82.6%) [20], northern Ethiopian pregnant women (59.4%) [15], in the central zone of Tigray Region, Ethiopia(85.67%) [21], and in Gondar Town, North West Ethiopia (64.9%) [22]. This might be due to the difference in the commitment of the providers in awareness-creation since the facilities are in the same country that is guided by the same minister of health and the availability of alternative hospitals in the town. It also lower than in South Africa (51%) [2] and Malaysia (56.2%) [16]. It might due to difference in research conducted time and using of updated guidelines.

Pregnant women who are educated are 75% more likely to have started ANC early compared to those who cannot read and write, and pregnant women who have a college diploma or higher are 85% more likely to have started ANC early compared to those who cannot read and write. This finding was consistent with the report of the study conducted in Dilla town governmental health institutions, illiteracy [18], Addis Ababa, Ethiopia, to eighth grade or below [13] and Debre Markos town, North West Ethiopia had, no formal education [11]. The studies found that higher levels of education of the mothers were less associated with late ANC initiation. This is probably due to the fact that education allows mothers to develop greater confidence to make better choices and to make decisions regarding their health as well as their children's. It is also more likely that educated women demand higher quality service and pay more attention to their health to ensure better health for themselves.

Pregnant women with an unplanned pregnancy (AOR = 11.290; 95%CI, 4.109–31.023) were 11.3 times more likely to start their ANC late compared to those women with planned pregnancies. It is consistent with the study conducted in Arba Minch Town and Arba Minch District, Gamo Gofa Zone, South Ethiopia with unplanned pregnancy [19], in Dilla town governmental health institutions, unplanned pregnancy [18], Debre Markos town [11] and Addis Ababa [13]. It is also consistent with the study conducted in Malaysia with unplanned pregnancy [16] and in South Africa unplanned pregnancy [2].

Women who reported arriving at the health facility in more than sixty (60) minutes were 8.28 times more likely to start ANC early than those who reported arriving in less than sixty (60) minutes. This finding was consistent with the report of study results conducted in Malaysia [15] and Cameroon [19].

Women with inadequate knowledge were four times less likely to begin ANC early than those with adequate knowledge. This finding was consistent with the report of study results conducted in the central zone of the Tigray Region, Ethiopia [21], and northern Ethiopian pregnant women [22]. Report of study conducted in Mekelle City [23] were also in line with result of study.

Pregnant women who had two children were six times more likely to begin ANC late than those who had three or more children. It is consistent with the report of study results conducted in Malaysia [16].

### Limitations of the study

The study only considers pregnant women attending public hospitals. As a result, a significant number of pregnant women who attended private health institutions and not visited health facility were overlooked. There is recall bias related to their gestational age, and we have tried to reduce it by asking about some memorable national holidays and reviewing their medical records information like ultrasound investigation.

## Conclusion

In the study area, late antenatal care initiation is a still high and serious public health issue. Educational status, unplanned pregnancy, time taken to get to a health facility, and inadequate knowledge of ANC services were affect the outcome variable. As a result, health care workers can provide ongoing health education on the need of starting antenatal care visits early to avoid unfavorable pregnancy outcomes. The hospital managers and the zonal health office should work together to raise knowledge about the advantages of starting ANC as soon as possible for both the mother and the fetus by considering the identified factor. Making the service more accessible to mothers who must travel long distances is also preferable.  

## Data Availability

Data will be available upon request from the corresponding author.

## Abbreviations

ANC: Antenatal care
CI: Confidence Interval
EDHS: Ethiopian demographic Health survey
WHO: World Health Organization
USD: USA Dollar
